# Association Between the Use of Proton Pump Inhibitors and Osteoporosis/Fracture: Nested Case—Control Studies Using a National Health Screening Cohort

**DOI:** 10.3390/jcm15103716

**Published:** 2026-05-12

**Authors:** Hyun Youk, Jeong Mi Park, Yoon Ji Kim, Ji Yeong Park, Hyo Geun Choi, Jung Woo Lee

**Affiliations:** 1 Department of Emergency Medicine, Wonju College of Medicine, Yonsei University, Wonju 26426, Republic of Korea; yhmentor@gmail.com; 2 MAIN Corp., Chuncheon 24341, Republic of Korea; silvia@gkmain.com; 3 Yonsei University Wonju Severance Christian Hospital, Wonju 26426, Republic of Korea; jeongmi815@kakao.com (J.M.P.); jzero110@naver.com (J.Y.P.); 4 Suseoseoulent Clinic, Seoul 06349, Republic of Korea; pupen@naver.com; 5 Mdanalytics, Seoul 06349, Republic of Korea; 6 Department of Orthopedic Surgery, Wonju College of Medicine, Yonsei University, Wonju 26426, Republic of Korea; 7 Yonsei Institute of Sports Science and Exercise Medicine, Wonju College of Medicine, Yonsei University, Wonju 26426, Republic of Korea; 8 Biobytes Inc., Chuncheon 24341, Republic of Korea

**Keywords:** proton pump inhibitors, osteoporosis, fractures, nested case-control study, propensity score weighting

## Abstract

**Background/Objectives**: Proton pump inhibitors (PPIs) are widely used for the treatment of acid-related disorders. However, there are concerns that PPI use may be associated with an increased risk of osteoporosis and fractures. This study aimed to investigate the association between PPI use and the risk of osteoporosis and fractures using national data. **Methods**: Two nested case—control studies using Korean National Health Insurance Service-Health Screening Cohort (514,866 participants, 2002–2019). Study I: 68,719 osteoporosis patients matched 1:1 with 68,719 controls by age, sex, income, region. Study II: 72,456 fracture patients matched 1:1 with 72,456 controls, stratified by fracture site (distal radius [n = 25,882], hip [n = 7753], spine [n = 38,821]). PPI use defined as prescription 1-year preceding index date: current users (≤30 days) and exposed group (31–365 days). Associations were analyzed using propensity score overlap-weighted logistic regression. **Results**: For osteoporosis, current PPI use showed odds ratio (OR) = 37.4 (95% confidence interval [CI] 33.3–42.1, *p* < 0.001); exposed group OR = 2.62 (95% CI 2.32–2.96, *p* < 0.001). Duration-dependent relationship observed: <30 days OR = 8.67, 30–180 days OR = 29.9, ≥180 days OR = 25.0. For fractures, current use associated with distal radius (OR = 37.4, 95% CI 28.8–48.7), hip (OR = 20.3, 95% CI 13.7–30.3), and spine (OR = 29.8, 95% CI 23.7–37.4). However, the exposed group showed no significant associations (distal radius OR = 1.24 *p* = 0.125; hip OR = 1.34 *p* = 0.174; spine OR = 1.26 *p* = 0.057). **Conclusions**: Current PPI use is strongly associated with increased odds of osteoporosis and fracture in the Asian population, with prominent duration—response relationship. Past PPI exposure showed no significant fracture risk. Healthcare providers should carefully assess bone health risks and consider lowest effective PPI doses.

## 1. Introduction

Osteoporosis is one of the most prevalent musculoskeletal disorders, characterized by reduced bone mineral density (BMD) and deterioration of bone microarchitecture, leading to increased fragility and fracture susceptibility [1]. The clinical burden of osteoporotic fractures remains substantial; in the United States, the estimated annual incidence rate of hospitalization for osteoporotic fractures was 995 per million persons in 2016 and 1114 per million persons in 2018 [2]. The pathophysiology of age-related bone loss involves distinct mechanisms: in postmenopausal women, rapid bone loss results from a negative bone remodeling balance due to estrogen deficiency, whereas in men, reduced bone formation is the primary driver [1]. In addition, various secondary causes contribute to the development of osteoporosis, including chronic diseases and concomitant medications.

Proton pump inhibitors (PPIs) are among the most widely prescribed medications worldwide, used primarily for the treatment of acid-related gastrointestinal disorders. In the United States, outpatient PPI prescriptions increased from 7.7% in 2012 to 9.7% in 2015, with the majority of users lacking a documented indication for use [3]. Given the accumulating evidence of adverse events associated with long-term PPI use [4], the American Gastroenterological Association has recommended prescribing PPIs at the lowest effective dose and for the shortest necessary duration [5]. A major concern is the potential impact of PPIs on bone health. PPIs suppress gastric acid secretion by inhibiting H+/K+ ATPase, which may impair intestinal calcium absorption and subsequently affect bone metabolism, although the precise mechanisms remain to be fully elucidated [6].

The relationship between PPI use and bone outcomes has been extensively investigated with somewhat inconsistent results. A systematic review and meta-analysis of observational studies reported no statistically significant effect of PPI use on mean annualized percent change in BMD (0.06; 95% CI −0.07 to 0.18) [7]. In contrast, a meta-analysis of 18 studies involving 244,109 fracture cases found that PPI use modestly increased the risk of hip fracture (RR = 1.26, 95% CI 1.16–1.36), spine fracture (relative risk [RR] = 1.58, 95% CI 1.38–1.82), and any-site fracture (RR = 1.33, 95% CI 1.15–1.54) [8]. An updated meta-analysis incorporating 32 studies further confirmed these associations, reporting increased risks of any-site fracture (hazard ratio [HR] 1.30, 95% CI 1.16–1.45), hip fracture (HR 1.22, 95% CI 1.15–1.31), spine fracture (HR 1.49, 95% CI 1.31–1.68), and osteoporosis (HR 1.23, 95% CI 1.06–1.42) [9]. Another meta-analysis of 24 studies with 2,103,800 participants specifically examining hip fractures also demonstrated a significant association (RR 1.20, 95% CI 1.14–1.28), with evidence of a dose-response relationship [10]. However, these meta-analyses have been limited by the predominant inclusion of data from Western populations, with only a small proportion of studies conducted in Asian populations [9]. Given the documented racial and ethnic disparities in BMD, osteoporosis prevalence, and fracture incidence across different populations [11,12], evidence from Asian populations is critically needed.

Therefore, we conducted two nested case-control studies using a large nationwide health screening cohort from South Korea to comprehensively investigate the associations between PPI use and the risk of osteoporosis and fractures in the hip, spine, and distal radius in an Asian population.

## 2. Methods

This study was approved by the ethics committee of Hallym University Sacred Heart Hospital on 5 November 2019 (IRB No: 2019-10-023). We utilized the Korean National Health Insurance Service-Health Screening Cohort data for this study, and a comprehensive explanation for this cohort is provided elsewhere [13].

### 2.1. Participant Recruitment

#### 2.1.1. Study I—Osteoporosis

**Figure 1:**
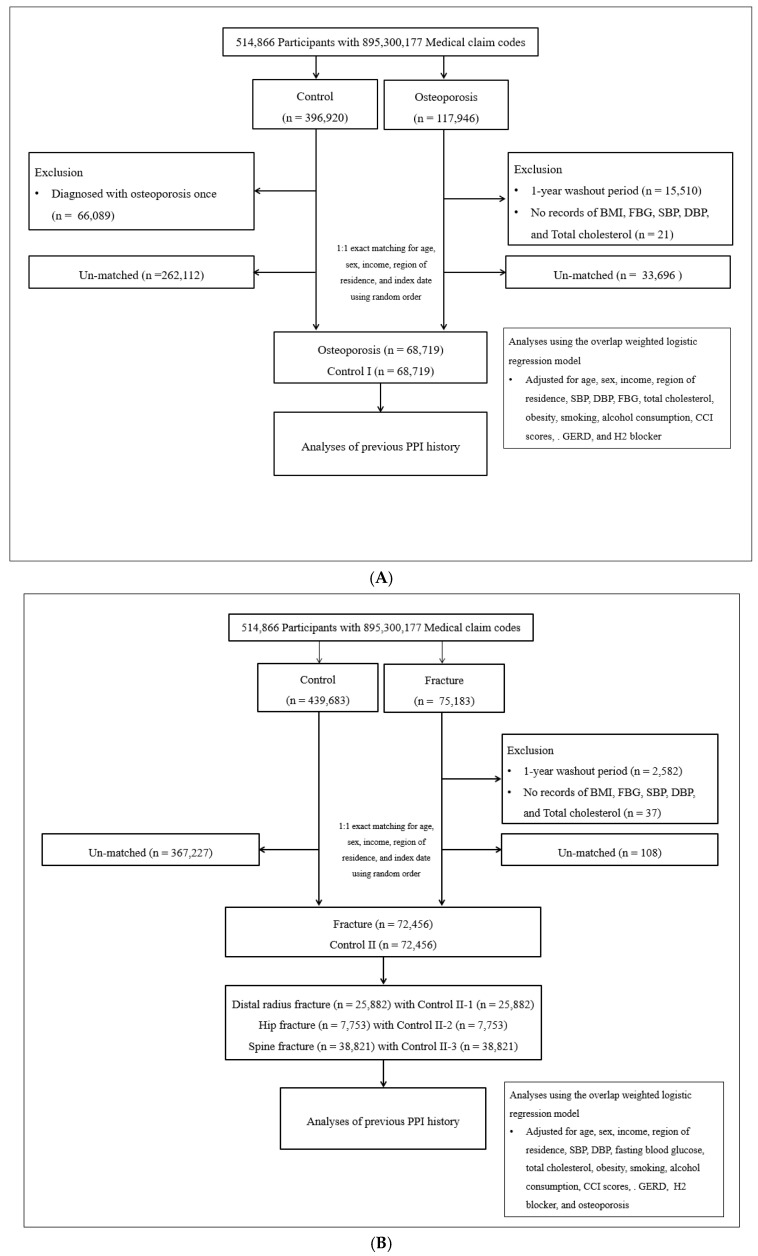
(**A**) A schematic illustration of the participant selection process that was used in the present study. Of a total of 514,866 participants, 68,719 osteoporosis participants were matched with 68,719 of control participants for age, sex, income, and region of residence. (**B**) A schematic illustration of the participant selection process that was used in the present study. Of a total of 514,866 participants, 72,456 of fracture participants were matched with 72,456 of control II participants for age, sex, income, and region of residence.

The participant selection process for Study I is detailed in Figure 1A. Among the 514,866 participants with 895,300,177 medical claim codes from 2002 through 2019, patients with osteoporosis (N = 117,946) and the control I group who were not diagnosed with osteoporosis between 2002 and 2019 (N = 396,920) were initially enrolled. Out of the 117,946 patients with osteoporosis, those who had a record of osteoporosis diagnosis in 2002 (1-year washout periods, N = 15,510) and those who had no records of body mass index (BMI), blood pressure, fasting blood glucose, and total cholesterol were excluded (N = 21). For the control I group, individuals who had been diagnosed with osteoporosis at least once were omitted (N = 66,089).

Participants with osteoporosis were matched in a 1:1 ratio with the control I group based on age, sex, income, and region of residence. The control I group was randomly selected to minimize the selection bias. The index date of each participant with osteoporosis was set as the time of treatment of osteoporosis. The index date of control I participants was set as the index date of their matched participant with osteoporosis. By doing this, each matched participant with osteoporosis has the same index date as control I participants. During the matching procedure, 33,696 participants with osteoporosis and 262,112 control I participants were excluded. Finally, 68,719 participants with osteoporosis and matched control I participants were included in Study I.

#### 2.1.2. Study II—Fractures

The participant selection process for Study II is detailed in Figure 1B. Out of 514,866 participants with 895,300,177 medical claim codes from 2002 through 2019, participants with fractures (N = 75,183) and the control II group who were not diagnosed with fractures (N = 439,683) were enrolled.

Among the 75,183 patients with fractures, those who had a record of fracture diagnosis in 2002 (1-year washout periods, N = 2582) and who had no records of BMI, blood pressure, fasting blood glucose, and total cholesterol were excluded (N = 37).

Participants with fractures were matched in a 1:1 ratio with the control II group based on age, sex, income, and region of residence. The control II group was randomly selected to minimize the selection bias. The index date of each participant with fractures was set as the time of treatment of fracture. The index date of control II participants was set as the index date of their matched fracture participants. By doing this, each matched participant with fractures has the same index date as control II participants. During the matching procedure, 108 participants with fractures and 367,227 control II participants were excluded. Finally, 72,456 participants with fractures and matched control II participants were included in Study II.

Among them, distal radius fracture (n = 25,882) with Control II-1 (n = 25,882) and Hip fracture (n = 7753) with Control II-2 (n = 7753) and Spine fracture (n = 38,821) with Control II-3 (n = 38,821) were 1:1 matched (Figure 1B).

### 2.2. Exposure

The use of PPI was defined as the prescription and duration within 1-year preceding the index date. PPI prescription was classified into 3 groups: (1) non-users, (2) current PPI users (prescribed at least once within 30 days), and (3) PPI-exposed individuals (prescribed at least once between 31 and 365 days).

### 2.3. Outcome

Osteoporosis was identified in participants who had diagnoses indicated by ICD-10 codes M80 (osteoporosis with pathological fracture), M81 (osteoporosis without pathological fracture), and M82 (osteoporosis in diseases classified elsewhere) between 2002 and 2019. From this group, we selected participants who underwent treatment for osteoporosis at least twice and those who were diagnosed with osteoporosis through Bone Densitometry using the dual-energy X-ray absorptiometry (DEXA) or DEXA CT scan (claim code: E7001-E7004), following our previous studies [14,15].

Regarding fractures, distal radius fracture was defined as a fracture of the lower end of the radius (ICD-10 codes: S525) [16]. Hip fracture was defined as a fracture of the head and neck of the femur (S720), a pertrochanteric fracture (S721), or a subtrochanteric fracture of the femur (S722) [17]. Spine fracture was defined as a fracture of a thoracic vertebra (S220) or a lumbar vertebra (S320) [17].

### 2.4. Study Covariates

Our study covariates included age, sex, income, region of residence, weight, smoking status, alcohol consumption status, systolic blood pressure (SBP), diastolic blood pressure (DBP), fasting blood glucose (FBG), total cholesterol, and Charlson Comorbidity Index (CCI) (Table 1).

Participants’ age was grouped into 5-year intervals: 40–44, 45–49 …, and 85+ years old, resulting in a total of 10 age groups. Income levels were stratified into five classes (ranging from class 1 [the lowest income] to class 5 [the highest income]). The region of residence was grouped into urban and rural areas. Smoking status was divided into the following three groups: non-smoker, past smoker, and current smoker. Alcohol consumption was categorized into two groups (<1 time a week vs. ≥1 time a week). Participants’ weights, as measured by BMI, were segmented into five groups: <18.5 (underweight), ≥18.5 to <23 (normal), ≥23 to <25 (overweight), ≥25 to <30 (obese I), and ≥30 (obese II). CCI is a widely used measure for assessing one’s disease burden, incorporating 17 comorbidities. This index ranges from 0 (no comorbidities) to 29 (multiple comorbidities) [18,19], which is given to each participant depending on one’s number of diseases and their severity. In our study, we excluded rheumatic disease in calculating the CCI score.

We also collected the following covariates: (1) the number of diagnoses of gastroesophageal reflux disease (GERD) (ICD-10 code: K21, treated ≥2 times and prescribed PPI for ≥2 weeks) for the 1 year before the index date and (2) the number of treatments for H2 blocker for 1 year before index date.

### 2.5. Statistical Analysis

We employed propensity score overlap weighting to ensure the covariate balance and enhance effective sample size. Propensity scores (PS) were derived via multivariable logistic regression that incorporates all study covariates. In calculating overlap weighting, which ranges from 0 to 1, participants were weighted based on the probability of 1-PS for cases and the probability of PS for controls [20,21,22]. The standardized difference was used to compare the differences in general characteristics between cases and controls (i.e., participants with osteoporosis vs. control I groups and participants with fractures and control II groups).

PS overlap weighted multivariable logistic regression analyses were used to investigate the associations between the use of PPIs and osteoporosis/fracture, of which the estimates were overlap weighted odds ratios (ORs) and corresponding 95% CI. In the analyses, crude (unadjusted) and overlap-weighted models (adjusted) were utilized. The latter model was adjusted for age, sex, income, region of residence, weight, smoking status, alcohol consumption status, SBP, DBP, FBG, total cholesterol, CCI scores, the number of diagnoses of GERD for the 1 year before the index date, and the number of treatments for H2 blocker for 1 year before index date.

We performed subgroup analyses for osteoporosis as follows: age groups (<60 vs. ≥60), sex (male vs. female), income groups (low vs. high), region of residence (urban vs. rural), weight (underweight, normal, overweight, and obese), smoking status (non-smoker vs. past and current smoker), alcohol consumption status (<1 time a week vs. ≥1 time a week), blood pressure (SBP < 140 and DBP < 90 vs. SBP ≥ 140 or DBP ≥ 90), FBG (<100 vs. ≥100 mg/dL), total cholesterol (<240 vs. ≥240 mg/dL), CCI scores (0, 1, and ≥2), GERD, and the use of H2 blocker.

All statistical analyses were two-tailed, and the statistical significance was set at *p* < 0.05. The SAS version 9.4 (SAS Institute Inc., Cary, NC, USA) was used for all statistical analyses.

## 3. Results

### 3.1. Study Participant Characteristics

Detailed characteristics of study participants before and after PS overlap weighting adjustment for each entity (osteoporosis, hip, spine, and distal radius fractures) are presented in Table 1 and Tables S1–S3. Regarding osteoporosis, most of the baseline characteristics were similar for osteoporosis and control I groups, except for PPI user and PPI exposure (Table 1). After PS overlap, 94.19% of patients with osteoporosis and 53.93% of control I patients were current PPI users, and 5.38% of patients with osteoporosis and 38.38% of control I patients were past PPI users (standardized difference = 1.04). The PPI prescription dates were longer in patients with osteoporosis than the control I group, both in duration of 30 to 180 days and over 180 days (standardized difference = 0.58).

### 3.2. Association Between PPI Use and Osteoporosis (Study I)

Our results regarding the association between PPI use (current PPI use and exposure to PPI) and osteoporosis are displayed in Table 2. After adjustment, compared to non-users, both current PPI use (OR 37.4, 95% CI 33.3 to 42.1; *p* < 0.001) and exposure to PPI (OR 2.62, 95% CI 2.32 to 2.96; *p* < 0.001) were significantly associated with a higher risk of osteoporosis.

Notably, our analysis showed the trend of duration–response relationship. The ORs after adjustment were estimated to be 8.67 (95% CI 7.69 to 9.77; *p* < 0.001) for < 30 days of exposure, 29.9 (95% CI 26.6 to 33.7; *p* < 0.001) for 30 to 180 days, and 25.0 (95% CI 22.2 to 28.1; *p* < 0.001) for 180 days and more.

### 3.3. Association Between PPI and Fractures in Hip, Spine, and Distal Radius (Study II)

Our results regarding the association between PPI use (current PPI use and exposure to PPI) and fractures in the distal radius, hip, and spine are displayed in Table 3, Table 4 and Table 5. After adjustment, compared to non-users, current PPI use was associated with an increased risk of distal radius fractures (OR 37.4, 95% CI 28.8 to 48.7; *p* < 0.001; Table 3), hip fractures (OR 20.3, 95% CI 13.7 to 30.3; *p* < 0.001; Table 4), and spine fractures (OR 29.8, 95% CI 23.7 to 37.4; *p* < 0.001; Table 5). In contrast, exposure to PPI showed non-significant results across all outcomes: distal radius fractures (OR 1.24, 95% CI 0.94 to 1.64; *p* = 0.125; Table 3), hip fractures (OR 1.34, 95% CI 0.88 to 2.05; *p* = 0.174; Table 4), and spine fractures (OR 1.26, 95% CI 0.99 to 1.60; *p* = 0.057; Table 5).

Similar to Study I, our analysis also found the trend of duration-response relationship for the fractures in the hip and spine. For the fractures in the hip, ORs after adjustment were estimated to be 7.65 (95% CI 5.08 to 11.5; *p* < 0.001) for <30 days of exposure, 15.2 (95% CI 10.1 to 22.7; *p* < 0.001) for 30 to 180 days, and 14.5 (95% CI 9.70 to 21.5; *p* < 0.001) for 180 days and more (Table 4). For the fractures in the spine, ORs after adjustment were estimated to be 12.2 (95% CI 9.73 to 15.4; *p* < 0.001) for <30 days of exposure, 22.3 (95% CI 17.7 to 28.0; *p* < 0.001) for 30 to 180 days, and 18.4 (95% CI 14.7 to 23.1; *p* < 0.001) for 180 days and more (Table 5).

### 3.4. Subgroup Analysis

**Figure 2:**
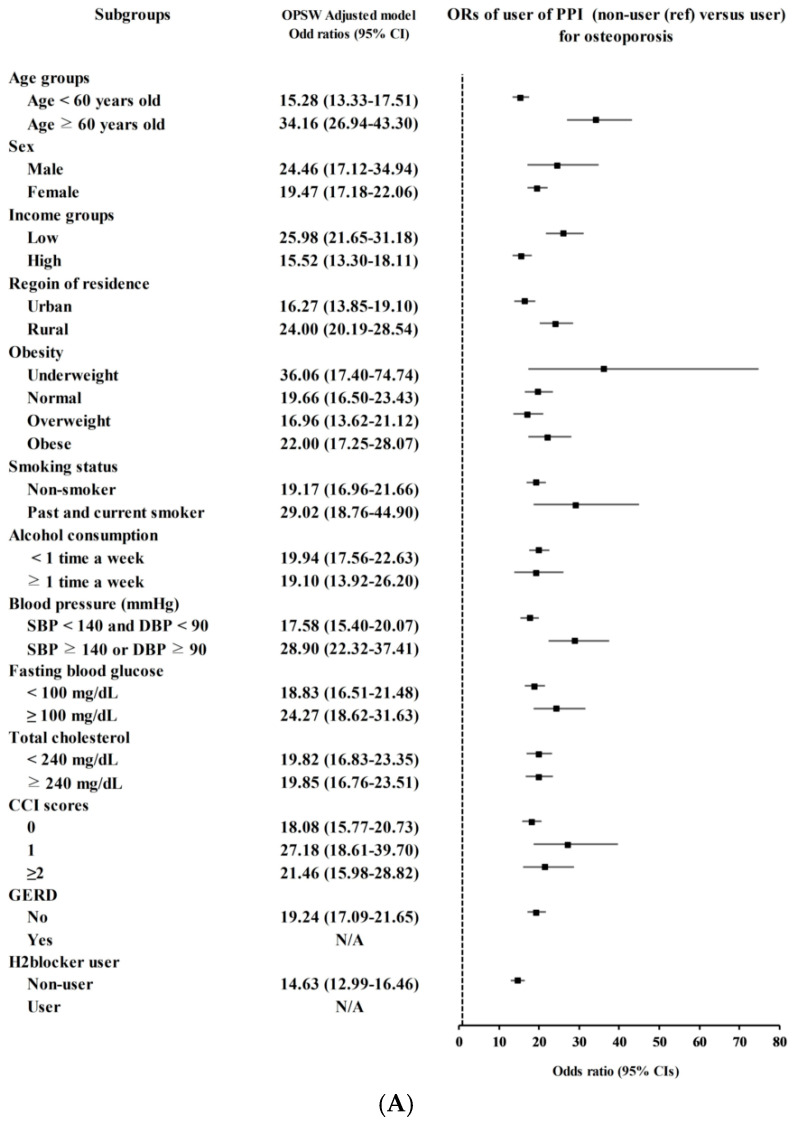

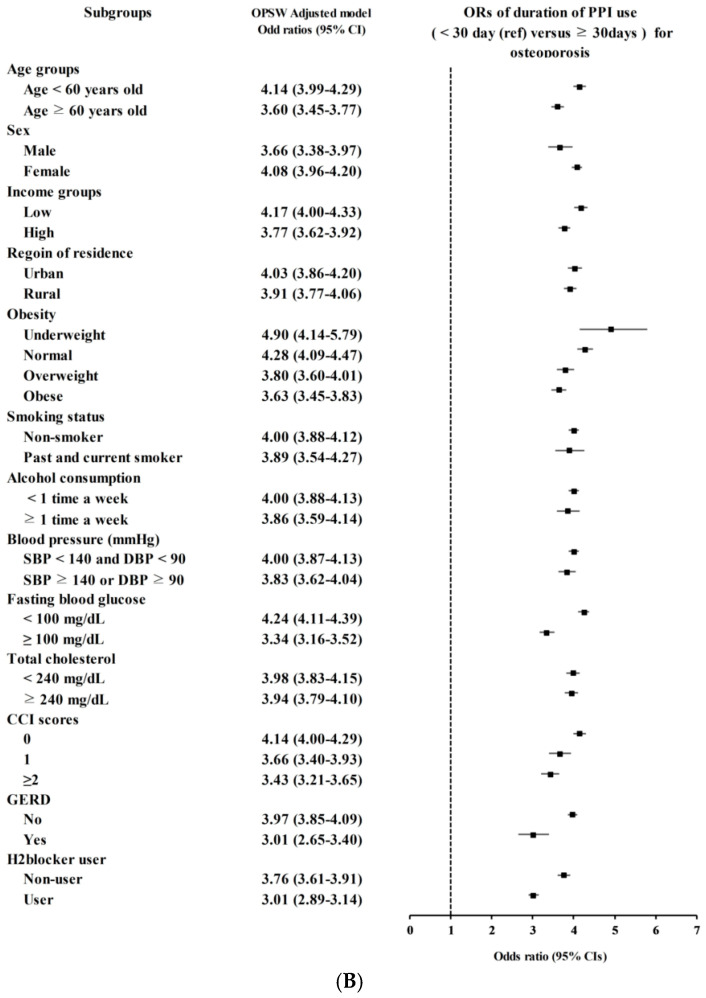
Forest plots of subgroup analyses for the association between proton pump inhibitor (PPI) use and osteoporosis. (**A**) Overlap propensity score-weighted odds ratios (ORs) for osteoporosis comparing PPI users versus non-users across subgroups defined by age, sex, income, region of residence, obesity, smoking status, alcohol consumption, blood pressure, fasting blood glucose, total cholesterol, Charlson Comorbidity Index (CCI) score, gastroesophageal reflux disease (GERD) status, and H2-blocker use. N/A indicates that odds ratios could not be estimated due to an insufficient number of PPI non-users in the subgroup. (**B**) Overlap propensity score-weighted odds ratios for osteoporosis comparing longer-duration PPI use (≥30 days) versus shorter-duration use (<30 days, reference) across the same subgroups. Squares represent point estimates and horizontal lines represent 95% confidence intervals. The vertical dashed line indicates the null value (OR = 1).

Results from subgroup analyses are illustrated in Figure 2. The observed association between PPI use and osteoporosis seemed to be moderated by age groups (<60 years old: OR 15.28, 95% CI 13.33 to 17.51 vs. ≥60 years old: OR 34.16, 95% CI 26.94 to 43.30), sex (male: OR 24.46, 95% CI 17.12 to 34.94 vs. female: OR 19.47, 95% CI 17.18 to 22.06), income groups (low: OR 25.98, 95% CI 21.65 to 31.18 vs. high: OR 15.52, 95% CI 13.30 to 18.11), region of residence (urban: OR 16.27, 95% CI 13.85 to 19.10 vs. rural: OR 24.00, 95% CI 20.19 to 28.54), weight (underweight: OR 36.06, 95% CI 17.40 to 74.74; normal: OR 19.66, 95% CI 16.50 to 23.43; overweight: OR 16.96, 95% CI 13.62 to 21.12; obese: OR 22.00, 95% CI 17.25 to 28.07), smoking status (non-smoker: OR 19.17, 95% CI 16.96 to 21.66 vs. past and current smoker: OR 29.02, 95% CI 18.76 to 44.90), and blood pressure (SBP < 140 and DBP < 90: OR 17.58, 95% CI 15.40 to 20.07 vs. SBP ≥ 140 or DBP ≥ 90: OR 28.90, 95% CI 22.32 to 37.41) (Figure 2A). However, for osteoporosis, the calculated ORs for PPI use of ≥30 days compared to <30 days did not show significant variation across various subgroups. Interestingly, the ORs were approximately 4.00 for all groups (Figure 2B).

## 4. Discussion

This observational study based on two nested case-control studies using a national health screening cohort of South Korea, incorporating data from 137,438 participants for Study I (osteoporosis) and 144,912 participants for Study II (fractures), investigated the association between the use of PPI and the risk of osteoporosis and fractures. Our findings from Study I suggested that the current use of PPI and exposure to PPI were associated with an increased risk of osteoporosis compared to non-users. Notably, longer use of PPI was found to be related to a higher risk of osteoporosis. However, regarding Study II for fractures, only the current use of PPI was associated with an elevated risk of fractures in the hip, spine, and distal radius than non-users while exposure to PPI showed non-significant results.

Numerous studies have been conducted that examined the potential links between PPI and the risk of bone diseases. Indeed, a systematic review and meta-analysis that included 32 studies conclusively reported their findings from observational studies that the use of PPI was moderately associated with osteoporosis and any site fractures [9]. However, this systematic review primarily relied on data from Western populations, with only 5 (15.6%) of the 32 included articles involving Asian participants (4 from Taiwan and 1 from South Korea). When considering the observed racial and ethnic differences in bone outcomes [12], our study contributed to providing valuable evidence on the association between the use of PPI and osteoporosis/fractures in an Asian population.

Our findings indicated that exposure to PPI was associated with a higher risk of osteoporosis than non-users even after fully adjusted (OR 2.62, 95% CI 2.32 to 2.96). The observed effect size was greater than that of a previous meta-analysis (pooled hazard ratio [HR] 1.23, 95% CI 1.06 to 1.42) [9]. Given that our study predominantly involved female individuals aged middle to older adulthood, the following findings from previous studies may support our results: (1) bone loss was faster in perimenopausal and postmenopausal Asian women than non-Hispanic white and black women [23] and (2) Asians exhibited the highest age-adjusted prevalence of osteoporosis and low bone mass regardless of sex [24]. Notably, our study found that the current use of PPI was strongly related to increased risk of osteoporosis, displaying a remarkable effect size of OR 37.4 (95% CI 33.3 to 42.1). While the abovementioned evidence may also contribute to the observed large effect size, we hypothesized that this might be due to confounding by indication. Specifically, indications for PPIs, such as their preventive usage alongside non-steroidal anti-inflammatory drugs (NSAIDs), might have influenced the observed effect size, given that the independent variable is the ‘current use of PPI’. Nonetheless, these findings carry significant clinical implications, suggesting that clinicians should be particularly alerted regarding the risk of osteoporosis among Asian female patients in middle to older adulthood, especially those who are using PPIs.

We also investigated the association between exposure to PPI and current use of PPI and risk of fractures. Our analysis revealed that exposure to PPI did not exhibit statistically significant associations with the fractures in the hip (OR 1.34, 95% CI 0.88 to 2.05), spine (OR 1.26, 95% CI 0.99 to 1.60), and distal radius (OR 1.24, 95% CI 0.94 to 1.64). Although these findings seemed to be inconsistent with a previous meta-analysis, which reported a HR of 1.30 (95% CI 1.16 to 1.45) for any-site fractures, a subgroup analysis for hip fractures by study location within the same study demonstrated the diminished effect size in Asian studies (HR 1.18, 95% CI 1.03 to 1.36) than European (HR 1.22, 95% CI 1.06 to 1.41) and North American (HR 1.25, 95% CI 1.14 to 1.37) studies [9]. Another meta-analysis that examined the association between PPI and hip fractures showed non-significant results in Asian subgroup analysis (RR 1.20, 95% CI 0.98–1.48) [10]. Of note, previous studies reported a lower incidence of fractures in the Asian population than in other race and ethnicity groups [11,25], which may potentially explain the non-significant results from the present study. Meanwhile, current use of PPI was also strongly associated with higher risks of the hip (OR 20.3, 95% CI 13.7–30.3), spine (OR 29.8, 95% CI 23.7 to 37.4), and distal radius (OR 37.4, 95% CI 28.8 to 48.7), which may also be due to confounding by indication as above. However, as indicated in a comprehensive review on this matter [6], the evidence on the association between PPI use and bone outcomes in the Asian population is still lacking, and therefore, our results should not be considered conclusive. Herein, clinicians should not dismiss the potential risk of fractures in individuals who have been exposed to PPI or are currently using PPI.

Although the biological mechanisms underlying the association between PPI use and adverse bone outcomes remain unclear, several potential pathways have been suggested. One hypothesized chronic pathway involves the suppression of gastric acid, which increases gastric pH and may subsequently reduce the solubility and intestinal absorption of dietary calcium salts [26]. Theoretically, such chronic malabsorption could trigger secondary hyperparathyroidism, potentially leading to accelerated bone resorption and a decline in BMD over time [27,28]. On the other hand, the pronounced association observed in our study—where current PPI use was strongly related to fracture risk while past exposure showed non-significant results—raises the possibility that acute physiological changes might also be involved [29,30]. Previous literature suggests that current PPI use could be associated with acute electrolyte imbalances, particularly hypomagnesemia and vitamin B12 deficiency, both of which can induce muscle weakness, peripheral neuropathy, or impaired balance [31,32]. These factors may acutely increase the risk of falls, leading to fractures even before a significant decline in BMD occurs [33]. Furthermore, in vitro evidence suggests that PPIs may directly inhibit vacuolar H+/K+ ATPase in human osteoclasts and osteoblasts, potentially disrupting the bone remodeling cycle shortly after the initiation of therapy [34].

Of note, our main analysis consistently showed the trend of duration-response relationship across all outcomes (osteoporosis, fractures in hip, spine, and distal radius). However, previous studies reported inconsistent results on the duration-response relationship. Subgroup analysis for hip fractures based on the duration of PPI usage in a prior meta-analysis did not indicate a significant difference in effect size (<1 year: HR 1.31, 95% CI 1.13 to 1.53 vs. ≥1 year: HR 1.37, 95% CI 1.26 to 1.49) [9]. Another study that employed meta-analysis also suggested a similar conclusion that longer PPI exposure might not be associated with a higher risk of hip fractures when used for over 1 year (<1 year: RR 1.21, 95% CI 1.16 to 1.25; 1~2 years: RR 1.23, 95% CI 1.07 to 1.42; >2 years: RR 1.24, 95% CI 1.10 to 1.40) [10]. These findings seemed to be in line with our results that effect sizes were smaller for PPI use <30 days compared to durations spanning 30 to 180 days, while >180 days did not exhibit a significant difference compared to 30 to 180 days. That is, we could hypothesize that a critical period for osteoporosis or fractures associated with PPI may exist between 30 to 180 days of PPI use. Moreover, when considering the existing evidence was limited to hip fractures, our study extended these observations across various outcomes, including osteoporosis and fractures in the hip, spine, and distal radius.

The results from subgroup analyses potentially identified the vulnerable population to osteoporosis in relation to PPI use. Notably, older age, male, low-income group, rural, underweight, past and current smokers, and hypertension were found to be associated with elevated risk of osteoporosis compared to their control group. These results could offer valuable insights since, to date, meta-analytic evidence concerning potential moderators for the association between PPI use and bone diseases has been limited to fractures [9]. Findings from our subgroup analysis suggested that healthcare providers encountering patients using PPIs could assess the individual’s susceptibility to osteoporosis by considering factors such as age, sex, income level, region of residence, weight, smoking status, and the presence of hypertension.

Several limitations should be noted in this study. First, the observational nature of our nested case–control design inherently precludes the establishment of causal relationships. While our results demonstrated substantial estimates and are supported by biologically plausible mechanisms [6], these findings must be strictly interpreted as epidemiological associations. Second, despite our efforts to adjust for potential confounding factors, including age, sex, income, region of residence, weight, smoking status, alcohol consumption status, SBP, DBP, FBG, total cholesterol, and CCI scores, we should acknowledge that residual confounding, particularly by indication, may have influenced our results. For example, indications for PPI use (e.g., concomitant administration with NSAIDs) might have affected the observed associations. Third, our dataset precluded the stratification of outcomes by specific PPI dosages or individual pharmacological agents. This is a crucial limitation, as different PPI formulations (e.g., omeprazole versus pantoprazole) possess distinct pharmacokinetic properties and acid-suppressive potencies that may differentially impact bone metabolism [35,36]. Furthermore, the lack of dosage information prevented us from evaluating potential dose-response gradients, such as comparing the risks associated with high-dose regimens versus standard maintenance therapy. Indeed, previous studies have demonstrated significant dose–response relationships and drug-specific variations in fracture risk, underscoring the clinical importance of these unmeasured variables [10]. Fourth, as our analysis was conducted retrospectively, there is a possibility of recall bias and misclassification of exposure or outcome variables. Fifth, the reliance on administrative claims data to define exposure and outcomes introduces the potential for misclassification bias. For instance, classifying individuals as “current users” based solely on prescription records does not guarantee actual medication adherence. Such non-adherence could lead to exposure misclassification, which may potentially attenuate or distort the true magnitude of the association. Lastly, our analysis did not account for anti-osteoporotic medication use (e.g., bisphosphonates, denosumab, or SERMs) at the individual level. As these medications are preferentially prescribed to individuals with established osteoporosis or high fracture risk [37,38,39], their uncontrolled effects may have influenced the observed associations. Future studies should incorporate anti-osteoporotic treatment as a covariate or conduct sensitivity analyses in untreated subgroups.

## 5. Conclusions

Despite these limitations, our study identified the association between the use of PPI and osteoporosis/fractures in the hip, spine, and distal radius in an Asian population. Our findings revealed that exposure to PPI was associated with an increased odds of osteoporosis while fractures showed non-significant results. Current PPI use displayed a remarkable association with all outcomes of osteoporosis and fractures. However, we must strictly emphasize that results in this observational study do not imply causality. These strong associations must be interpreted cautiously, and potential residual confounding, such as confounding by indication, should be considered. Nevertheless, healthcare providers should not overlook the potential risk of osteoporosis or fractures in individuals who have been prescribed PPIs, particularly in elderly women. Of note, our analysis based on the duration of PPI use suggested that there might be a critical period of increased risk occurring between 30 to 180 days of PPI use. To overcome the inherent limitations of observational designs and formally establish causality, future prospective studies and Mendelian randomization studies are strongly warranted to validate these potential associations.

## Figures and Tables

**Table 1:** General characteristics of participants propensity score overlap weighting adjustment.

Characteristic	After PS Overlap Weighting Adjustment			Before PS Overlap Weighting Adjustment		
	Osteoporosis (n, %)	Control I (n, %)	Standardized Difference	Osteoporosis (n, %)	Control I (n, %)	Standardized Difference
Total participants (n, %)						
Age (%)			0.00			0.00
40–44	508 (1.53)	508 (1.53)		1035 (1.51)	1035 (1.51)	
45–49	2533 (7.64)	2533 (7.64)		5198 (7.56)	5198 (7.56)	
50–54	5959 (17.98)	5959 (17.98)		12,347 (17.97)	12,347 (17.97)	
55–59	7573 (22.85)	7573 (22.85)		15,727 (22.89)	15,727 (22.89)	
60–64	6312 (19.05)	6312 (19.05)		13,127 (19.10)	13,127 (19.10)	
65–69	3535 (10.67)	3535 (10.67)		7333 (10.67)	7333 (10.67)	
70–74	3436 (10.37)	3436 (10.37)		7138 (10.39)	7138 (10.39)	
75–79	2199 (6.64)	2199 (6.64)		4566 (6.64)	4566 (6.64)	
80–84	899 (2.71)	899 (2.71)		1858 (2.70)	1858 (2.70)	
85+	186 (0.56)	186 (0.56)		390 (0.57)	390 (0.57)	
Sex (%)			0.00			0.00
Male	6155 (18.57)	6155 (18.57)		12,924 (18.81)	12,924 (18.81)	
Female	26,984 (81.43)	26,984 (81.43)		55,795 (81.19)	55,795 (81.19)	
Income (%)			0.00			0.00
1 (lowest)	6208 (18.73)	6208 (18.73)		12,898 (18.77)	12,898 (18.77)	
2	5000 (15.09)	5000 (15.09)		10,363 (15.08)	10,363 (15.08)	
3	5424 (16.37)	5424 (16.37)		11,266 (16.39)	11,266 (16.39)	
4	6745 (20.35)	6745 (20.35)		13,995 (20.37)	13,995 (20.37)	
5 (highest)	9764 (29.46)	9764 (29.46)		20,197 (29.39)	20,197 (29.39)	
Region of residence (%)			0.00			0.00
Urban	14,039 (42.36)	14,039 (42.36)		29,051 (42.28)	29,051 (42.28)	
Rural	19,101 (57.64)	19,101 (57.64)		39,668 (57.72)	39,668 (57.72)	
Weight † (%)			0.00			0.17
Underweight	890 (2.69)	890 (2.69)		2106 (3.06)	1628 (2.37)	
Normal	12,379 (37.35)	12,379 (37.35)		27,879 (40.57)	23,473 (34.16)	
Overweight	8760 (26.43)	8760 (26.43)		17,915 (26.07)	18,145 (26.40)	
Obese I	9991 (30.15)	9991 (30.15)		18,978 (27.62)	22,427 (32.64)	
Obese II	1120 (3.38)	1120 (3.38)		1841 (2.68)	3046 (4.43)	
Smoking status (%)			0.00			0.03
Non-smoker	29,127 (87.89)	29,127 (87.89)		60,576 (88.15)	59,979 (87.28)	
Past smoker	1790 (5.40)	1790 (5.40)		3761 (5.47)	3778 (5.50)	
Current smoker	2223 (6.71)	2223 (6.71)		4382 (6.38)	4962 (7.22)	
Alcohol consumption status (%)			0.00			0.03
<1 time a week	27,138 (81.89)	27,138 (81.89)		56,507 (82.23)	55,792 (81.19)	
≥1 time a week	6001 (18.11)	6001 (18.11)		12,212 (17.77)	12,927 (18.81)	
SBP (Mean, SD)	126.53 (13.11)	126.53 (12.58)	0.00	125.31 (18.58)	127.84 (18.54)	0.14
DBP (Mean, SD)	78.08 (8.15)	78.08 (7.85)	0.00	77.73 (11.67)	78.49 (11.39)	0.07
FBG (Mean, SD)	97.91 (27.38)	97.91 (17.73)	0.00	95.47 (31.60)	100.90 (33.47)	0.17
Total cholesterol (Mean, SD)	202.41 (27.82)	202.41 (27.19)	0.00	200.96 (39.12)	203.72 (39.71)	0.07
CCI score (Mean, SD)	1.06 (1.17)	1.06 (1.25)	0.00	1.08 (1.72)	1.04 (1.77)	0.02
GERD for 1 year before index date (Mean, SD)	0.37 (0.89)	0.37 (1.09)	0.00	0.52 (1.79)	0.29 (1.29)	0.15
The number of treatments for H2 blocker for 1 year before index date (Mean, SD)	22.36 (31.27)	22.36 (40.27)	0.00	29.20 (58.25)	17.68 (48.42)	0.22
User of PPI (n, %)			1.04			1.05
Non-user	143 (0.43)	2551 (7.70)		283 (0.41)	5398 (7.86)	
Current user	31,214 (94.19)	17,871 (53.93)		64,838 (94.35)	36,853 (53.63)	
Past user	1783 (5.38)	12,717 (38.38)		3598 (5.24)	26,468 (38.52)	
Duration of PPI use (n, %)			0.58			0.60
Non-user	143 (0.43)	2551 (7.70)		283 (0.41)	5398 (7.86)	
<30 days	4526 (13.66)	9370 (28.27)		8965 (13.05)	19,556 (28.46)	
30 to 180 days	13,175 (39.76)	8352 (25.20)		27,113 (39.45)	17,027 (24.78)	
≥180 days	15,296 (46.16)	12,866 (38.82)		32,358 (47.09)	26,738 (38.91)	

Abbreviations: CCI, Charlson Comorbidity Index; SBP, systolic blood pressure; DBP, diastolic blood pressure; FBG, fasting blood glucose; PS, propensity score; GERD, gastroesophageal reflux disease; † weight (BMI, body mass index, kg/m^2^) was categorized as <18.5 (underweight), ≥18.5 to <23 (normal), ≥23 to <25 (overweight), ≥25 to <30 (obese I), and ≥30 (obese II).

**Table 2:** Crude and overlap propensity-score-weighted odd ratios of proton pump inhibitor (ref: non-user) for osteoporosis.

Characteristic	N of Osteoporosis	N of Control I	Odds Ratios for Osteoporosis (95% Confidence Interval)			
	(exposure/total, %)	(exposure/total, %)	Crude	*p*-value	Overlap weighted model †	*p*-value
User of PPI						
Current PPI use	64,838/68,719 (94.4)	36,853/68,719 (53.6)	33.5 (29.7–37.8)	<0.001 *	37.4 (33.3–42.1)	<0.001 *
PPI exposed	3598/68,719 (5.2)	26,468/68,719 (38.5)	2.59 (2.29–2.93)	<0.001 *	2.62 (2.32–2.96)	<0.001 *
Duration of PPI use						
<30 days	8965/68,719 (13.1)	19,556/68,719 (28.5)	8.73 (7.73–9.86)	<0.001 *	8.67 (7.69–9.77)	<0.001 *
30 to 180 days	27,113/68,719 (39.5)	17,027/68,719 (24.8)	30.3 (26.9–34.2)	<0.001 *	29.9 (26.6–33.7)	<0.001 *
≥180 days	32,358/68,719 (47.1)	26,738/68,719 (38.91)	23.0 (20.4–26.0)	<0.001 *	25.0 (22.2–28.1)	<0.001 *

* Significance at *p* < 0.05. † Adjusted for age, sex, income, region of residence, SBP, DBP, FBG, total cholesterol, obesity, smoking, alcohol consumption, CCI scores, GERD, and H2 blocker.

**Table 3:** Crude and overlap propensity-score-weighted odd ratios of proton pump inhibitor (ref: non-user) for distal radius fracture.

Characteristic	N of Distal Radius Fracture	N of Control II-1	Odd Ratios for Distal Radius Fracture (95% Confidence Interval)			
	(exposure/total, %)	(exposure/total, %)	Crude	*p*-value	Overlap weighted model †	*p*-value
User of PPI						
Current PPI use	25,346/25,882 (97.9)	16,458/25,882 (63.6)	30.6 (23.6–39.8)	<0.001 *	37.4 (28.8–48.7)	<0.001 *
PPI exposed	477/25,882 (1.8)	8250/25,882 (31.9)	1.15 (0.87–1.52)	0.322	1.24 (0.94–1.64)	0.125
Duration of PPI use						
<30 days	4744/25,882 (18.3)	4647/25,882 (18.0)	20.3 (15.6–26.4)	<0.001 *	20.5 (15.7–26.7)	<0.001 *
30 to 180 days	6928/25,882 (26.8)	5742/25,882 (22.2)	24.0 (18.4–31.2)	<0.001 *	23.6 (18.1–30.7)	<0.001 *
≥180 days	14,151/25,882 (54.7)	14,319/25,882 (55.3)	19.6 (15.1–25.5)	<0.001 *	19.7 (15.1–25.6)	<0.001 *

* Significance at *p* < 0.05. † Adjusted for age, sex, income, region of residence, SBP, DBP, FBG, total cholesterol, obesity, smoking, alcohol consumption, CCI scores, GERD, H2 blocker, and osteoporosis.

**Table 4:** Crude and overlap propensity-score-weighted odd ratios of proton pump inhibitor (ref: non-user) for hip fracture.

Characteristic	N of Hip Fracture	N of Control II-2	Odd Ratios for Hip Fracture (95% Confidence Interval)			
	(exposure/total, %)	(exposure/total, %)	Crude	*p*-value	Overlap weighted model †	*p*-value
User of PPI						
Current PPI use	7565/7753 (97.6)	5550/7753 (71.6)	19.0 (12.5–28.7)	<0.001 *	20.3 (13.7–30.3)	<0.001 *
PPI exposed	164/7753 (2.1)	1869/7753 (24.1)	1.22 (0.78–1.90)	0.378	1.34 (0.88–2.05)	0.174
Duration of PPI use						
<30 days	484/7753 (6.2)	906/7753 (11.7)	7.43 (4.84–11.4)	<0.001 *	7.65 (5.08–11.5)	<0.001 *
30 to 180 days	1322/7753 (17.1)	1204/7753 (15.5)	15.3 (10.0–23.3)	<0.001 *	15.2 (10.1–22.7)	<0.001 *
≥180 days	5923/7753 (76.4)	5309/7753 (68.48)	15.5 (10.2–23.5)	<0.001 *	14.5 (9.70–21.5)	<0.001 *

* Significance at *p* < 0.05. † Adjusted for age, sex, income, region of residence, SBP, DBP, FBG, total cholesterol, obesity, smoking, alcohol consumption, CCI scores, GERD, H2 blocker, and osteoporosis.

**Table 5:** Crude and overlap propensity score weighted odd ratios of proton pump inhibitor (ref: non user) for spine fracture.

Characteristic	N of Spine Fracture	N of Control II-3	Odd Ratios for Spine Fracture (95% Confidence Interval)			
	(exposure/total, %)	(exposure/total, %)	Crude	*p*-value	Overlap weighted model †	*p*-value
User of PPI						
Current PPI use	38,138/38,821 (98.2)	27,088/38,821 (69.8)	30.1 (23.6–38.4)	<0.001 *	29.8 (23.7–37.4)	<0.001 *
PPI exposed	615/38,821 (1.6)	10,280/38,821 (26.5)	1.28 (0.99–1.65)	0.061	1.26 (0.99–1.60)	0.057
Duration of PPI use						
<30 days	3084/38,821 (7.9)	5053/38,821 (13.0)	13.0 (10.2–16.6)	<0.001 *	12.2 (9.73–15.4)	<0.001 *
30 to 180 days	8918/38,821 (23.0)	7269/38,821 (18.7)	26.1 (20.5–33.4)	<0.001 *	22.3 (17.7–28.0)	<0.001 *
≥180 days	26,751/38,821 (68.9)	25,046/38,821 (64.5)	22.8 (17.8–29.0)	<0.001 *	18.4 (14.7–23.1)	<0.001 *

* Significance at *p* < 0.05. † Adjusted for age, sex, income, region of residence, SBP, DBP, FBG, total cholesterol, obesity, smoking, alcohol consumption, CCI scores, GERD, H2 blocker, and osteoporosis.

## Data Availability

The data that support the findings of this study are not publicly available due to institutional data governance policies governing the Korean National Health Insurance Service (NHIS) database. The statistical analysis code is available from the corresponding author (J.W.L.) upon reasonable request.

## Supplementary Materials

The following supporting information can be downloaded at: https://www.mdpi.com/article/10.3390/jcm15103716/s1, Table S1: Baseline characteristics of participants in the distal radius fracture cohort (Study II-1) before and after propensity score overlap weighting; Table S2: Baseline characteristics of participants in the hip fracture cohort (Study II-2) before and after propensity score overlap weighting; Table S3: Baseline characteristics of participants in the spine fracture cohort (Study II-3) before and after propensity score overlap weighting.
