# Supplementary material for: Association Between the Use of Proton Pump Inhibitors and Osteoporosis/Fracture: Nested Case—Control Studies Using a National Health Screening Cohort
Source: J Clin Med. 2026 May 12;15(10):3716. doi: 10.3390/jcm15103716 (PMC13207836; doi:10.3390/jcm15103716)
Supplement: Supplementary file 1 [file jcm-15-03716-s001.zip › S1 table.pdf]

S1 table General Characteristics of Participants propensity score overlap weighting adjustment

Characteristics	After PS Overlap weighting adjustment			Before PS Overlap weighting adjustment		
	Distal radius fracture (n, %)	Control II-1 (n, %)	Standardized Difference	Distal radius fracture (n, %)	Control II-1 (n, %)	Standardized Difference
Total participants (n, %)						
Age (%)			0.00			0.00
40-44	106 (0.84)	106 (0.84)		213 (0.82)	213 (0.82)	
45-49	502 (3.97)	502 (3.97)		1,018 (3.93)	1,018 (3.93)	
50-54	1,476 (11.66)	1,476 (11.66)		2,991 (11.56)	2,991 (11.56)	
55-59	2,542 (20.09)	2,542 (20.09)		5,179 (20.01)	5,179 (20.01)	
60-64	2,455 (19.40)	2,455 (19.40)		5,020 (19.40)	5,020 (19.40)	
65-69	2,125 (16.79)	2,125 (16.79)		4,353 (16.82)	4,353 (16.82)	
70-74	1,671 (13.20)	1,671 (13.20)		3,443 (13.30)	3,443 (13.30)	
75-79	1,090 (8.62)	1,090 (8.62)		2,247 (8.68)	2,247 (8.68)	
80-84	506 (4.00)	506 (4.00)		1,041 (4.02)	1,041 (4.02)	
85+	182 (1.44)	182 (1.44)		377 (1.46)	377 (1.46)	

Sex (%)			0.00			0.00
Male	3,099 (24.49)	3,099 (24.49)		6,320 (24.42)	6,320 (24.42)	
Female	9,556 (75.51)	9,556 (75.51)		19,562 (75.58)	19,562 (75.58)	
Income (%)			0.00			0.00
1 (lowest)	2,285 (18.05)	2,285 (18.05)		4,679 (18.08)	4,679 (18.08)	
2	1,724 (13.62)	1,724 (13.62)		3,534 (13.65)	3,534 (13.65)	
3	2,036 (16.09)	2,036 (16.09)		4,154 (16.05)	4,154 (16.05)	
4	2,672 (21.11)	2,672 (21.11)		5,459 (21.09)	5,459 (21.09)	
5 (highest)	3,939 (31.13)	3,939 (31.13)		8,056 (31.13)	8,056 (31.13)	
Region of residence (%)			0.00			0.00
Urban	5,259 (41.55)	5,259 (41.55)		10,768 (41.60)	10,768 (41.60)	
Rural	7,396 (58.45)	7,396 (58.45)		15,114 (58.40)	15,114 (58.40)	
Obesity † (%)			0.00			0.09
Underweight	309 (2.44)	309 (2.44)		627 (2.42)	632 (2.44)	
Normal	4,506 (35.60)	4,506 (35.60)		9,614 (37.15)	8,799 (34.00)	

Overweight	3,457 (27.32)	3,457 (27.32)		7,084 (27.37)	7,010 (27.08)	
Obese I	3,974 (31.40)	3,974 (31.40)		7,841 (30.30)	8,412 (32.50)	
Obese II	409 (3.23)	409 (3.23)		716 (2.77)	1,029 (3.98)	
Smoking status (%)			0.00			0.01
Nonsmoker	10,584 (83.63)	10,584 (83.63)		21,655 (83.67)	21,655 (83.67)	
Past smoker	587 (4.64)	587 (4.64)		1,175 (4.54)	1,230 (4.75)	
Current smoker	1,484 (11.73)	1,484 (11.73)		3,052 (11.79)	2,997 (11.58)	
Alcohol consumption (%)			0.00			0.04
<1 time a week	10,365 (81.90)	10,365 (81.90)		21,007 (81.16)	21,408 (82.71)	
≥1 time a week	2,290 (18.10)	2,290 (18.10)		4,875 (18.84)	4,474 (17.29)	
SBP (Mean, SD)	126.73 (13.11)	126.73 (11.80)	0.00	126.50 (18.59)	127.06 (17.02)	0.03
DBP (Mean, SD)	78.17 (8.23)	78.17 (7.48)	0.00	78.64 (11.83)	77.78 (10.64)	0.08
FBG (Mean, SD)	99.63 (29.44)	99.64 (17.33)	0.00	97.88 (35.81)	100.96 (27.77)	0.10
Total cholesterol (Mean, SD)	202.53 (28.35)	202.53 (27.43)	0.00	202.91 (39.92)	201.94 (39.25)	0.02
CCI score (Mean, SD)	0.96 (1.10)	0.96 (1.16)	0.00	1.01 (1.64)	0.91 (1.60)	0.06

GERD for 1 year before index date (Mean, SD)	0.59 (1.52)	0.59 (1.46)	0.00	0.60 (2.22)	0.57 (2.04)	0.01
The number of treatments for H2 blocker for 1 year before index date (Mean, SD)	29.75 (43.83)	29.75 (46.34)	0.00	30.79 (64.18)	28.91 (65.21)	0.03
Osteoporosis (n, %)			0.00			0.18
No	7,618 (60.20)	7,618 (60.20)		14,425 (55.73)	16,687 (64.47)	
Yes	5,037 (39.80)	5,037 (39.80)		11,457 (44.27)	9,195 (35.53)	
User of PPI (n, %)			0.96			0.97
Non-user	29 (0.23)	564 (4.46)		59 (0.23)	1,174 (4.54)	
Current user	12,391 (97.91)	8,064 (63.72)		25,346 (97.93)	16,458 (63.59)	
Past user	236 (1.86)	4,026 (31.82)		477 (1.84)	8,250 (31.88)	
Duration of PPI use (n, %)			0.29			0.30
Non-user	29 (0.23)	564 (4.46)		59 (0.23)	1,174 (4.54)	
< 30 days	2,361 (18.66)	2,267 (17.91)		4,744 (18.33)	4,647 (17.95)	
30 to 180 days	3,383 (26.73)	2,857 (22.57)		6,928 (26.77)	5,742 (22.19)	
≥ 180 days	6,882 (54.38)	6,968 (55.06)		14,151 (54.68)	14,319 (55.32)	

Abbreviations: CCI, Charlson Comorbidity Index; SBP, Systolic blood pressure; DBP, Diastolic blood pressure; FBG. Fasting blood glucose; PS, Propensity score; GERD, Gastroesophageal reflux disease;

† Obesity (BMI, body mass index, kg/m^2) was categorized as < 18.5 (underweight), ≥ 18.5 to < 23 (normal), ≥ 23 to < 25 (overweight), ≥ 25 to < 30 (obese I), and ≥ 30 (obese II)
