# Supplementary material for: Association Between the Use of Proton Pump Inhibitors and Osteoporosis/Fracture: Nested Case—Control Studies Using a National Health Screening Cohort
Source: J Clin Med. 2026 May 12;15(10):3716. doi: 10.3390/jcm15103716 (PMC13207836; doi:10.3390/jcm15103716)
Supplement: Supplementary file 1 [file jcm-15-03716-s001.zip › S2 table.pdf]

S2 table General Characteristics of Participants propensity score overlap weighting adjustment

Characteristics	After PS Overlap weighting adjustment			Before PS Overlap weighting adjustment		
	Hip fracture (n, %)	Control II-2 (n, %)	Standardized Difference	Hip fracture (n, %)	Control II-2 (n, %)	Standardized Difference
Total participants (n, %)						
Age (%)			0.00			0.00
40-44	16 (0.44)	16 (0.44)		32 (0.41)	32 (0.41)	
45-49	66 (1.86)	66 (1.86)		137 (1.77)	137 (1.77)	
50-54	127 (3.57)	127 (3.57)		272 (3.51)	272 (3.51)	
55-59	209 (5.87)	209 (5.87)		454 (5.86)	454 (5.86)	
60-64	258 (7.22)	258 (7.22)		575 (7.42)	575 (7.42)	
65-69	405 (11.35)	405 (11.35)		897 (11.57)	897 (11.57)	
70-74	579 (16.22)	579 (16.22)		1,282 (16.54)	1,282 (16.54)	
75-79	769 (21.56)	769 (21.56)		1,689 (21.79)	1,689 (21.79)	
80-84	685 (19.20)	685 (19.20)		1,470 (18.96)	1,470 (18.96)	
85+	453 (12.71)	453 (12.71)		945 (12.19)	945 (12.19)	

Sex (%)			0.00			0.00
Male	1,581 (44.32)	1,581 (44.32)		3,485 (44.95)	3,485 (44.95)	
Female	1,986 (55.68)	1,986 (55.68)		4,268 (55.05)	4,268 (55.05)	
Income (%)			0.00			0.00
1 (lowest)	748 (20.97)	748 (20.97)		1,620 (20.90)	1,620 (20.90)	
2	444 (12.44)	444 (12.44)		963 (12.42)	963 (12.42)	
3	518 (14.53)	518 (14.53)		1,128 (14.55)	1,128 (14.55)	
4	673 (18.87)	673 (18.87)		1,469 (18.95)	1,469 (18.95)	
5 (highest)	1,184 (33.19)	1,184 (33.19)		2,573 (33.19)	2,573 (33.19)	
Region of residence (%)			0.00			0.00
Urban	1,331 (37.33)	1,331 (37.33)		2,898 (37.38)	2,898 (37.38)	
Rural	2,235 (62.67)	2,235 (62.67)		4,855 (62.62)	4,855 (62.62)	
Obesity † (%)			0.00			0.12
Underweight	169 (4.73)	169 (4.73)		448 (5.78)	294 (3.79)	
Normal	1,318 (36.94)	1,318 (36.94)		2,953 (38.09)	2,755 (35.53)	

Overweight	898 (25.17)	898 (25.17)		1,896 (24.46)	1,987 (25.63)	
Obese I	1,065 (29.87)	1,065 (29.87)		2,211 (28.52)	2,447 (31.56)	
Obese II	117 (3.29)	117 (3.29)		245 (3.16)	270 (3.48)	
Smoking status (%)			0.00			0.10
Nonsmoker	2,649 (74.28)	2,649 (74.28)		5,625 (72.55)	5,844 (75.38)	
Past smoker	260 (7.29)	260 (7.29)		530 (6.84)	613 (7.91)	
Current smoker	657 (18.43)	657 (18.43)		1,598 (20.61)	1,296 (16.72)	
Alcohol consumption (%)			0.00			0.01
<1 time a week	2,784 (78.06)	2,784 (78.06)		6,004 (77.44)	6,041 (77.92)	
≥1 time a week	782 (21.94)	782 (21.94)		1,749 (22.56)	1,712 (22.08)	
SBP (Mean, SD)	131.67 (13.38)	131.67 (11.73)	0.00	132.90 (19.99)	130.78 (17.01)	0.11
DBP (Mean, SD)	79.68 (8.11)	79.68 (7.27)	0.00	80.93 (12.31)	78.64 (10.55)	0.20
FBG (Mean, SD)	105.05 (29.10)	105.05 (22.59)	0.00	108.31 (49.99)	104.05 (31.13)	0.10
Total cholesterol (Mean, SD)	198.18 (27.38)	198.18 (26.74)	0.00	200.66 (42.83)	196.10 (38.93)	0.11
CCI score (Mean, SD)	1.72 (1.25)	1.72 (1.45)	0.00	2.20 (2.18)	1.35 (1.87)	0.42

GERD for 1 year before index date (Mean, SD)	0.63 (1.49)	0.63 (1.52)	0.00	0.67 (2.35)	0.60 (2.15)	0.03
The number of treatments for H2 blocker for 1 year before index date (Mean, SD)	46.15 (54.42)	46.15 (60.90)	0.00	54.06 (89.60)	39.24 (81.27)	0.17
Osteoporosis (n, %)			0.00			0.19
No	2,236 (62.69)	2,236 (62.69)		4,531 (58.44)	5,226 (67.41)	
Yes	1,331 (37.31)	1,331 (37.31)		3,222 (41.56)	2,527 (32.59)	
User of PPI (n, %)			0.73			0.77
Non-user	12 (0.35)	147 (4.13)		24 (0.31)	334 (4.31)	
Current user	3,470 (97.30)	2,596 (72.78)		7,565 (97.58)	5,550 (71.59)	
Past user	84 (2.35)	824 (23.10)		164 (2.12)	1,869 (24.11)	
Duration of PPI use (n, %)			0.31			0.34
Non-user	12 (0.35)	147 (4.13)		24 (0.31)	334 (4.31)	
< 30 days	250 (7.01)	395 (11.08)		484 (6.24)	906 (11.69)	
30 to 180 days	659 (18.49)	549 (15.38)		1,322 (17.05)	1,204 (15.53)	
≥ 180 days	2,645 (74.16)	2,476 (69.41)		5,923 (76.40)	5,309 (68.48)	

Abbreviations: CCI, Charlson Comorbidity Index; SBP, Systolic blood pressure; DBP, Diastolic blood pressure; FBG. Fasting blood glucose; PS, Propensity score; GERD, Gastroesophageal reflux disease;

† Obesity (BMI, body mass index, kg/m^2) was categorized as < 18.5 (underweight), ≥ 18.5 to < 23 (normal), ≥ 23 to < 25 (overweight), ≥ 25 to < 30 (obese I), and ≥ 30 (obese II)
