# Supplementary material for: Association Between the Use of Proton Pump Inhibitors and Osteoporosis/Fracture: Nested Case—Control Studies Using a National Health Screening Cohort
Source: J Clin Med. 2026 May 12;15(10):3716. doi: 10.3390/jcm15103716 (PMC13207836; doi:10.3390/jcm15103716)
Supplement: Supplementary file 1 [file jcm-15-03716-s001.zip › S3 table.pdf]

S3 table General Characteristics of Participants propensity score overlap weighting adjustment

Characteristics	After PS Overlap weighting adjustment			Before PS Overlap weighting adjustment		
	Spine fracture (n, %)	Control II-3 (n, %)	Standardized Difference	Spine fracture (n, %)	Control II-3 (n, %)	Standardized Difference
Total participants (n, %)						
Age (%)			0.00			0.00
40-44	70 (0.39)	70 (0.39)		142 (0.37)	142 (0.37)	
45-49	316 (1.78)	316 (1.78)		658 (1.69)	658 (1.69)	
50-54	823 (4.62)	823 (4.62)		1,735 (4.47)	1,735 (4.47)	
55-59	1,496 (8.40)	1,496 (8.40)		3,201 (8.25)	3,201 (8.25)	
60-64	2,298 (12.90)	2,298 (12.90)		5,012 (12.91)	5,012 (12.91)	
65-69	3,038 (17.06)	3,038 (17.06)		6,645 (17.12)	6,645 (17.12)	
70-74	3,479 (19.54)	3,479 (19.54)		7,656 (19.72)	7,656 (19.72)	
75-79	3,344 (18.78)	3,344 (18.78)		7,351 (18.94)	7,351 (18.94)	
80-84	2,117 (11.89)	2,117 (11.89)		4,616 (11.89)	4,616 (11.89)	
85+	829 (4.65)	829 (4.65)		1,805 (4.65)	1,805 (4.65)	

Sex (%)			0.00			0.00
Male	5,720 (32.12)	5,720 (32.12)		12,480 (32.15)	12,480 (32.15)	
Female	12,090 (67.88)	12,090 (67.88)		26,341 (67.85)	26,341 (67.85)	
Income (%)			0.00			0.00
1 (lowest)	3,461 (19.43)	3,461 (19.43)		7,550 (19.45)	7,550 (19.45)	
2	2,328 (13.07)	2,328 (13.07)		5,062 (13.04)	5,062 (13.04)	
3	2,687 (15.09)	2,687 (15.09)		5,828 (15.01)	5,828 (15.01)	
4	3,645 (20.46)	3,645 (20.46)		7,946 (20.47)	7,946 (20.47)	
5 (highest)	5,690 (31.95)	5,690 (31.95)		12,435 (32.03)	12,435 (32.03)	
Region of residence (%)			0.00			0.00
Urban	6,174 (34.67)	6,174 (34.67)		13,524 (34.84)	13,524 (34.84)	
Rural	11,636 (65.33)	11,636 (65.33)		25,297 (65.16)	25,297 (65.16)	
Obesity † (%)			0.00			0.04
Underweight	565 (3.17)	565 (3.17)		1,286 (3.31)	1,199 (3.09)	
Normal	6,181 (34.71)	6,181 (34.71)		13,694 (35.27)	13,161 (33.90)	

Overweight	4,574 (25.68)	4,574 (25.68)		9,853 (25.38)	10,074 (25.95)	
Obese I	5,844 (32.82)	5,844 (32.82)		12,627 (32.53)	12,874 (33.16)	
Obese II	646 (3.63)	646 (3.63)		1,361 (3.51)	1,513 (3.90)	
Smoking status (%)			0.00			0.04
Nonsmoker	14,288 (80.22)	14,288 (80.22)		30,881 (79.55)	31,426 (80.95)	
Past smoker	1,033 (5.80)	1,033 (5.80)		2,343 (6.04)	2,223 (5.73)	
Current smoker	2,489 (13.97)	2,489 (13.97)		5,597 (14.42)	5,172 (13.32)	
Alcohol consumption (%)			0.00			0.03
<1 time a week	14,396 (80.83)	14,396 (80.83)		31,077 (80.05)	31,721 (81.71)	
≥1 time a week	3,415 (19.17)	3,415 (19.17)		7,744 (19.95)	7,100 (18.29)	
SBP (Mean, SD)	129.72 (13.12)	129.72 (11.52)	0.00	129.59 (19.15)	129.94 (17.18)	0.02
DBP (Mean, SD)	79.07 (7.94)	79.07 (7.28)	0.00	79.73 (11.79)	78.43 (10.66)	0.12
FBG (Mean, SD)	100.96 (28.03)	100.96 (17.21)	0.00	98.74 (34.62)	102.93 (29.48)	0.13
Total cholesterol (Mean, SD)	200.90 (26.84)	200.90 (27.50)	0.00	202.84 (39.95)	198.93 (39.98)	0.10
CCI score (Mean, SD)	1.35 (1.20)	1.35 (1.30)	0.00	1.54 (1.94)	1.19 (1.77)	0.19

GERD for 1 year before index date (Mean, SD)	0.71 (1.52)	0.71 (1.67)	0.00	0.84 (2.71)	0.61 (2.17)	0.09
The number of treatments for H2 blocker for 1 year before index date (Mean, SD)	43.59 (50.68)	43.59 (57.41)	0.00	52.91 (85.88)	36.01 (75.49)	0.21
Osteoporosis (n, %)			0.00			0.41
No	9,146 (51.35)	9,146 (51.35)		16,133 (41.56)	23,838 (61.40)	
Yes	8,664 (48.65)	8,664 (48.65)		22,688 (58.44)	14,983 (38.60)	
User of PPI (n, %)			0.81			0.84
Non-user	37 (0.21)	612 (3.43)		68 (0.18)	1,453 (3.74)	
Current user	17,465 (98.06)	12,661 (71.09)		38,138 (98.24)	27,088 (69.78)	
Past user	309 (1.73)	4,537 (25.48)		615 (1.58)	10,280 (26.48)	
Duration of PPI use (n, %)			0.29			0.32
Non-user	37 (0.21)	612 (3.43)		68 (0.18)	1,453 (3.74)	
< 30 days	1,580 (8.87)	2,186 (12.27)		3,084 (7.94)	5,053 (13.02)	
30 to 180 days	4,289 (24.08)	3,360 (18.87)		8,918 (22.97)	7,269 (18.72)	
≥ 180 days	11,904 (66.84)	11,652 (65.42)		26,751 (68.91)	25,046 (64.52)	

Abbreviations: CCI, Charlson Comorbidity Index; SBP, Systolic blood pressure; DBP, Diastolic blood pressure; FBG. Fasting blood glucose; PS, Propensity score; GERD, Gastroesophageal reflux disease;

† Obesity (BMI, body mass index, kg/m^2) was categorized as < 18.5 (underweight), ≥ 18.5 to < 23 (normal), ≥ 23 to < 25 (overweight), ≥ 25 to < 30 (obese I), and ≥ 30 (obese II)
